# Evolution of brain network dynamics in neurodevelopment

**DOI:** 10.1162/NETN_a_00001

**Published:** 2017-02-01

**Authors:** Lucy R. Chai, Ankit N. Khambhati, Rastko Ciric, Tyler M. Moore, Ruben C. Gur, Raquel E. Gur, Theodore D. Satterthwaite, Danielle S. Bassett

**Affiliations:** Department of Bioengineering, University of Pennsylvania, Philadelphia, PA 19104 USA; Brain Behavior Laboratory, Department of Psychiatry, University of Pennsylvania, Philadelphia, PA 19104 USA; Department of Electrical & Systems Engineering, University of Pennsylvania, Philadelphia, PA 19104 USA

**Keywords:** Neurodevelopment, Executive function, Energy, Entropy, Matrix factorization, Subgraph, Flexibility

## Abstract

Cognitive function evolves significantly over development, enabling flexible control of human behavior. Yet, how these functions are instantiated in spatially distributed and dynamically interacting networks, or *graphs,* that change in structure from childhood to adolescence is far from understood. Here we applied a novel machine-learning method to track continuously overlapping and time-varying subgraphs in the brain at rest within a sample of 200 healthy youth (ages 8–11 and 19–22) drawn from the Philadelphia Neurodevelopmental Cohort. We uncovered a set of subgraphs that capture surprisingly integrated and dynamically changing interactions among known cognitive systems. We observed that subgraphs that were highly expressed were especially transient, flexibly switching between high and low expression over time. This transience was particularly salient in a subgraph predominantly linking frontoparietal regions of the executive system, which increases in both expression and flexibility from childhood to young adulthood. Collectively, these results suggest that healthy development is accompanied by an increasing precedence of executive networks and a greater switching of the regions and interactions subserving these networks.

## AUTHOR SUMMARY

Our ability to thoughtfully engage with the world around us changes appreciably as we transition from childhood to adulthood. Yet, how our brains develop to enable that change remains far from understood. Here we used network science—traditionally applied to the study of social networks like Facebook or Twitter—and machine learning to show that growing cognitive abilities are accompanied by greater flexibility of brain regions within distributed networks. This flexibility is greatest in the executive system, which is critical for higher-order cognitive functions and increases in expression and flexibility from childhood to young adulthood. These results suggest that healthy development is facilitated by an increasing precedence of executive networks and a greater switching of the regions and interactions subserving these networks.

## Introduction

Healthy human behavior requires flexibility to adapt existing neurophysiological processes to meet evolving task demands. A quintessential architecture that facilitates such adaptation is modularity (Felix & Wagner, 1998; Kirschner & Gerhart, 1998): Theoretically, sets of interconnected brain regions can alter their behavior in responses to the environment, without inducing unwanted or even pathological changes in other sets (Sporns & Betzel, 2016). Recent empirical work has supported these theoretical notions by identifying putative functional modules in the brain that map directly onto known cognitive systems (Power et al., 2011; Salvador, Suckling, Schwarzbauer, & Bullmore, 2005; Yeo et al., 2011), including the auditory, motor, default mode, and attention systems. A complementary line of inquiry has simultaneously demonstrated that putative functional modules can reconfigure over time as healthy human participants engage in training paradigms to learn new skills (Bassett et al., 2011; Bassett, Yang, Wymbs, & Grafton, 2015) or engage in task-switching paradigms requiring frequent changes in cognitive effort (Braun et al., 2015). Together, these studies point to large-scale brain networks as important units of cognitive function that dynamically integrate with and segregate from one another to enable human behavior (Cole, Bassett, Power, Braver, & Petersen, 2014; Mattar, Cole, Thompson-Schill, & Bassett, 2015).

The notion that large-scale brain networks form a repertoire whose integration is manipulated to meet task demands suggests several interesting questions. Are these systems engaged independently over time? Are there fundamental constraints on how much a system can be engaged in relation to other systems? Or are there constraints on how transiently a system can be engaged? These questions are particularly relevant to cognitive neuroscience, where evidence suggests that language, memory, and learning are supported by transient network-level control processes characteristic of executive function (Bassett et al., 2015; Braun et al., 2015; Chen et al., 2013; Cole et al., 2013; Fedorenko & Thompson-Schill, 2014). Moreover, they lend a critical point to recent evidence in developmental neuroscience demonstrating that the transience of brain states increases over development, in proportion to increases in executive performance (Medaglia et al., 2015). These studies support the notion that the transient engagement of higher-order cognitive systems—particularly executive systems—may be critical to understanding healthy cognition (Yeo et al., 2015) and its development from the nascent architecture of childhood to the developed architecture of young adulthood. Yet, gaining this understanding will require tools that can describe to what degree different systems are engaged at a given time, and how that engagement changes over time. Unfortunately, the current tools to study cognitive systems are either agnostic to temporal dynamics (e.g., common clustering techniques, including modularity maximization) or impose strict constraints on how systems can be engaged (e.g., hard-partitioning algorithms). Thus, although conceptual frameworks are beginning to be posited for the dynamic manipulation of cognitive modules (Robinson, Atlas, & Wager, 2015), progress in confirming these predictions has been stymied by fundamental insufficiencies in the neuroscientist’s computational toolset.

To address these limitations, we applied a dynamic machine-learning approach that utilizes parts-based matrix decomposition techniques to examine how the temporal properties of the resting brain network architecture develop in youth. Intuitively, matrix decomposition of a functional connectivity pattern yields (1) a dictionary of subgraphs overlapping in space and time, and (2) corresponding continuous time-dependent coefficients quantifying the expression of each subgraph. These time-dependent coefficients exist on a per-subject, per-time-window basis. As compared to hard-partitioning schemes, the advantage of this method is that it provides information about brain network dynamics in a continuous, overlapping manner in space and time, rather than discrete partitions. Furthermore, due to the parts-based nature of the technique, we obtained subgraphs that resembled localized features of large-scale brain networks, rather than generalized versions of the overall network (Lee & Seung, 1999). We applied this technique to a neurodevelopmental cohort to address several hypotheses. First, due to the parts-based nature of the method, we hypothesized that our dynamic subgraphs correspond to time-dependent interactions among well-known cognitive systems that are capable of directly modulating behavior. Second, we hypothesized that the magnitude and the temporal persistence of subgraph expression are differentially modulated by age. Third, we predicted that transient flexibility would be maximal in subgraphs that included fronto-parietal regions known to be involved in executive function.

**Figure 1.**
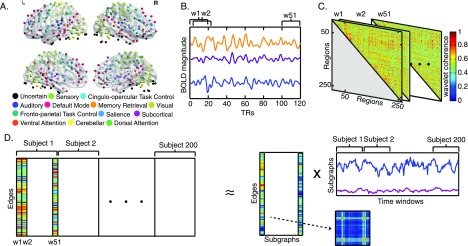
Schematic overview of the approach. (A) Resting-state fMRI BOLD signals were obtained from 264 functional regions of interest in cortical and subcortical areas, spanning 13 cognitive systems (Power et al., 2011). (B) Each regional BOLD signal was divided into 51 time windows, each 20 repetition times (TRs) in duration, with 90% overlap. (C) We computed the wavelet coherence between each pair of regional BOLD signals for every time window to obtain a multilayer network in which brain regions were treated as network nodes, and window-specific estimates of coherence were treated as layer-specific network edges. (D) We next unfolded the unique connections of the multilayer network and concatenated the data for all subjects (left). We then used a nonnegative matrix factorization approach, which decomposes the concatenated matrix into a matrix *W* of subgraphs and a matrix *H* of time-dependent coefficients that quantify the level of expression in each time window for each subgraph (right).

To address our hypotheses, we acquired resting-state functional magnetic resonance imaging (fMRI) data from 200 healthy subjects drawn from the Philadelphia Neurodevelopmental Cohort (PNC; see Methods) (Satterthwaite et al., 2014). Half of these subjects were between 8 and 11 years of age, and the other half were between 19 and 22 years of age. Although the fMRI scans were collected from a total of 780 adolescent participants in the PNC, we focused on the 100 youngest and 100 oldest participants, due to limitations in computational memory. After constructing dynamic functional connectivity matrices (see Methods; Figures 1A–1C), we performed matrix decomposition using a nonnegative matrix factorization technique (Kim & Park, 2007). We obtained a set of network subgraphs and time-dependent coefficients that quantified the level of expression for each subgraph (Figure 1D). As we describe below, we observed that our method reproduced several known properties of resting-state networks, but also provided new information from the temporally and spatially continuous nature of the subgraphs identified. We observed that the subgraphs captured extensive integration among cognitive systems, and that highly expressed subgraphs were more prone to change in expression over time, suggesting that subgraphs are either transiently or stably expressed on the basis of metabolic or energetic demands. Finally, we observed differences in the expression patterns of a subgraph involving the frontal and parietal cortices between the child group and the young adult group, suggesting that changes in network expression and network flexibility support executive function over development.

## RESULTS

### Integrated Nature of Subgraphs

The nonnegative matrix factorization approach uncovered a set of overlapping subgraphs, as well as the corresponding continuous time-dependent coefficients for each subgraph and subject. On the basis of our parameter optimization scheme (see Supplementary Information, Chai et al., 2017), we uncovered ten dynamical subgraphs over a 200-subject population of children and young adults. In the following sections, we first characterize the architecture of each subgraph, which captures the unique pattern of connections among the 264 regions (see Methods and Figure 1D), and then focus on the dynamical expression of these subgraphs over time and over subjects.

**Figure 2.**
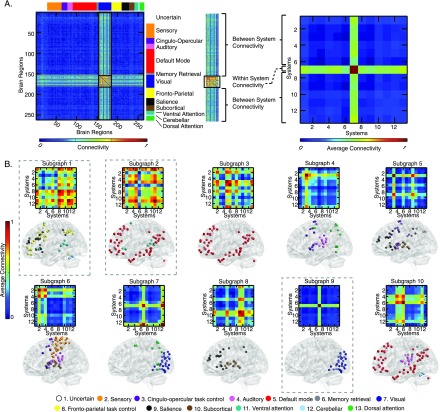
Mapping of subgraphs to cognitive systems. (A) From non-negative matrix factorization, we obtained a set of ten subgraphs, each capturing a unique pattern of connections among the 264 cortical and subcortical areas that was significantly expressed during the resting-state scan. Here we show an example of computing the within-system connectivity and between-system connectivity for the visual system in the ninth subgraph. From the raw subgraph (left), the within-system connectivity is computed as the average of the edge weights between nodes within a system (e.g., visual–visual edges), and the between-system connectivity is computed as the average of the edge weights between nodes from two different systems (e.g., visual–sensory edges). This approach allows us to summarize the relationship between the subgraph structure and the cognitive systems expressed in each subgraph in a 13 × 13 matrix (right). (B). We computed the average within-system and between-system connectivities for the 13 cognitive systems in each subgraph, normalizing the color bar between 0 and 1. We then compared the connectivity in each subgraph to permuted null graphs to identify which cognitive systems were significantly expressed in each subgraph (see Methods). The significantly expressed systems are depicted in brain volume renderings (the matrices themselves are not thresholded by significance). We observed that many subgraphs are distributed in nature, capturing interactions among multiple cognitive systems (e.g., Subgraphs 1 and 2), while others are localized in nature (e.g., Subgraph 9).

We first asked whether these ten dynamic subgraphs corresponded to time-dependent interactions among previously studied cognitive systems. To address this question, we used a partitioning of the 264 brain regions into 13 cognitive systems determined *a priori* (Power et al., 2011), including the visual, auditory, motor, default mode, salience, frontoparietal, cingulo-opercular, and attention systems; see Figure 1A. Next, for each subgraph, we computed the within-system connectivity and between-system connectivity of the cognitive systems (see Methods and Figure 2A). This procedure produced a 13 × 13 matrix of interactions among the cognitive systems expressed by each subgraph. Such a coarse-graining approach offers a simple summary of the mapping between subgraph architecture and cognitive function. While all systems are expressed to some extent due to the continuous nature of the matrix factorization approach—for simplicity and visualization purposes we aimed to pick out those systems that were the most important contributors in each 13 × 13 subgraph by using a permutation test to retain the magnitudes of within-system and between-system subgraph connectivity that were stronger than expected (see Methods).

We observed that the matrix decomposition yielded subgraphs that corresponded to interactions within and between previously defined cognitive systems, as we had anticipated because of the parts-based nature of the approach. Moreover, each subgraph was differentially distributed over cognitive systems (Figure 2B), and we used this information to parsimoniously categorize subgraphs into *simple* and more *complex* subgraphs. Pictorially, simple subgraphs capture interactions within a single cognitive system; for example, the visual regions in the ninth subgraph depict strong within-system connectivity. In contrast, complex subgraphs capture interactions among two or more cognitive systems. For example, the first subgraph—which we will hereafter refer to as the *executive subgraph* —displays high expression across a number of regions in frontal and parietal cortex that are associated with executive function. Specifically, we observed significant expression in the frontoparietal task control, salience, and ventral attention systems.

In general, we observed that the cognitive systems relating to higher-level cognitive functions (task control, attention, memory, and salience) tended to act in concert with each other, or with sensorimotor systems (auditory, visual, and sensory) in complex subgraphs. Specifically, we observed that the average number of significantly expressed systems in the subgraphs was consistently greater than 1 (one-sample *t*-test: *t*_(9)_ = 4.38, *p* = 0.002). The presence of these complex subgraphs is particularly interesting, because it suggests that cognitive systems are unlikelyare unlikely to act in isolation from one another. For example, we observed patterns of interactions between default mode regions and ventral attention regions; these regions have traditionally been considered separate, although some recent literature has motivated a reevaluation of this assumption (Gu et al., 2015; Karahanoğlu & Van De Ville, 2015; Power et al., 2011). The fact that these subgraphs do not map in a one-to-one manner to individual cognitive systems suggests that cognitive systems do not function as distinct entities over short timescales. Instead, cognitive systems closely interact with one another in transient processes that collectively produce the complex landscape of brain dynamics that supports cognition.

### Subgraphs Differ in Expression and Dynamics

**Figure 3.**
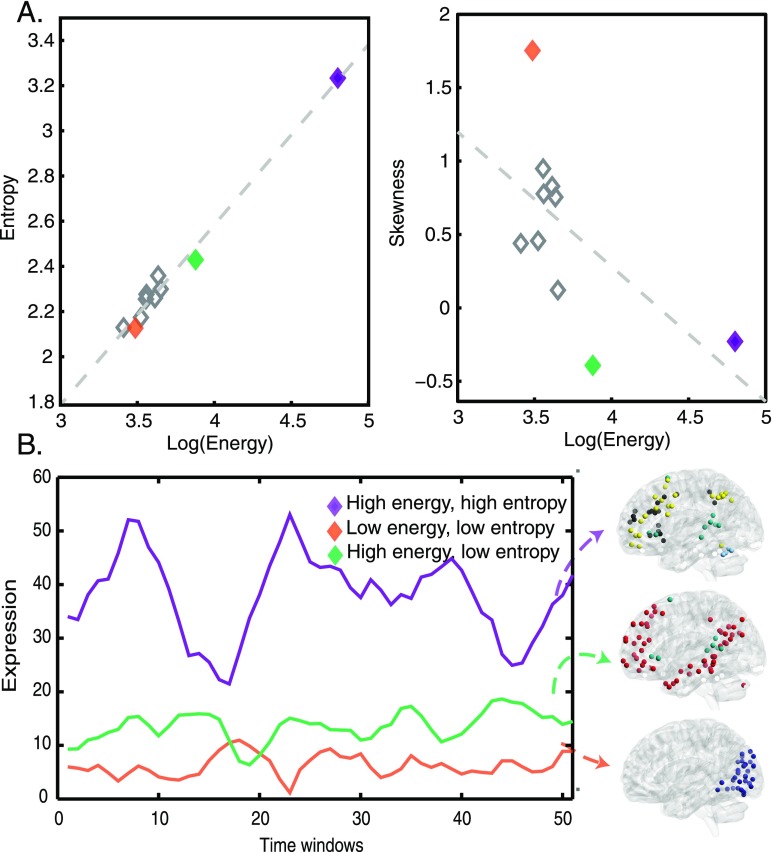
Energy and entropy of subgraph expression. (A) For each subgraph, we averaged the energy and entropy of the corresponding time-dependent coefficients across all subjects. We observed a high correlation between the log of subgraph energy and entropy (Pearson correlation coefficient *r* = 0.99, *p* < 0.001). The default mode subgraph (shown in green) is an interesting deviation from the trend (left). We observed an interesting trend between the localities of the subgraphs (as measured by the skewness of the subgraph strength; see Methods) and subgraph energy, suggesting that more distributed subgraphs have higher energy, and more localized subgraphs have lower energy (right). (B) Examples of a high-energy, high-entropy signal (executive); a low-energy, low-entropy signal (visual); and a high-energy, low-entropy signal (default mode) for a representative subject (left), and the cognitive systems associated with these signals (right). In general, we observed that subgraphs with high-energy, high-entropy expression patterns tended to involve multiple distributed interacting cognitive systems, while subgraphs with low-energy, low-entropy expression patterns tended to involve fewer localized cognitive systems.

Up to this point, we have only considered the network topology of functional subgraphs expressed collectively among the cohort of children and young adult subjects. We now consider the time-varying expression of subgraphs—or subgraph dynamics—across individuals. Using the time-varying subgraph expression coefficients computed by the NMF technique, we asked two questions: (1) Do highly expressed subgraphs fluctuate more rapidly in time?, and (2) Do subgraph dynamics predict features of subgraph topology? To measure how strongly a subgraph is expressed over time windows, we computed the *energy* of subgraph expression for temporal coefficients of each subgraph for each subject. Similarly, to measure how transiently the subgraph was expressed, we also computed the entropy of subgraph expression using a histogram estimator (see Methods). We note that the *entropy* metric is also highly correlated with the temporal derivative (the absolute value of the first order difference between adjacent coefficients; see Supplementary Information, Chai et al., 2017), an indication that the distribution of expression coefficients is related to a subgraph’s underlying dynamics. We observed that the entropy and logarithm of energy of the subgraphs were highly correlated (Pearson correlation coefficient: *r* = 0.99, *p* < 0.001, Figure 3A): strongly expressed subgraphs have a greater tendency to change in their levels of expression, with high uncertainty, while weakly expressed subgraphs have a greater tendency to remain stable in their levels of expression, with less uncertainty. (Note that a slight deviation from this trend was observed in the subgraph capturing default mode regions, which had high energy but relatively lower entropy, suggesting that this subgraph is highly expressed but less prone to change in expression; Figure 3A.)

Interestingly, we observed a trend in that the subgraphs with high-energy, high-entropy expression patterns tended to be spatially distributed and engage multiple interacting cognitive systems. For example, the subgraph with the highest energy and entropy corresponded to the executive subgraph, which captures interactions among a number of higher-order cognitive systems. In contrast, the subgraphs with low energy, low entropy expression patterns (e.g., the visual system) tended to be spatially localized and engage fewer cognitive systems (Figure 3B; see also Figure 1A, for mapping to cognitive systems, and Methods). Using spatial skewness of the 13 × 13 subgraph matrix as a metric for the locality of the subgraphs (see Methods), we observed a trend in which skewness was inversely related to the energy (Pearson correlation coefficient *r* = −0.59, *p* = 0.069), suggesting that the more distributed subgraphs have higher energy, while the more localized subgraphs have lower energy. Taken together, these results suggest the presence of a constraint on brain dynamics: strongly expressed subgraphs are likely to be more volatile in their expression and incorporate many cognitive systems, while weakly expressed subgraphs are likely to be more stable in their expression and incorporate fewer cognitive systems.

### Executive Function and Neurodevelopment

Given that prior findings have reported changes in functional brain network structure relating to executive function throughout childhood and adolescence, we were interested in how the executive subgraph—which captures interactions among many higher-order regions of the executive system—changes in expression levels and stability of expression as children mature. In the previous section, we noted the distinctively high energy and entropy of the executive subgraph compared to the remaining subgraphs. We further hypothesized that the energy and entropy of the executive subgraph might differ between the group of 100 children and the group of 100 young adults.

To that end, we performed an *a priori* analysis using the continuous time-dependent coefficients corresponding to the executive subgraph (Subgraph 1). Using the time-dependent coefficients as a measure of subgraph expression, we first corrected for differences in each individual’s baseline executive subgraph expression by standardizing each subject’s time-dependent coefficients by dividing by the mean. We note that this approach critically enabled us to compare relative rather than absolute weights. We then statistically compared the standardized energy and entropy of subgraph expression between the group of 100 children (ages 8–11) and the group of 100 young adults (ages 19–22).

**Figure 4.**
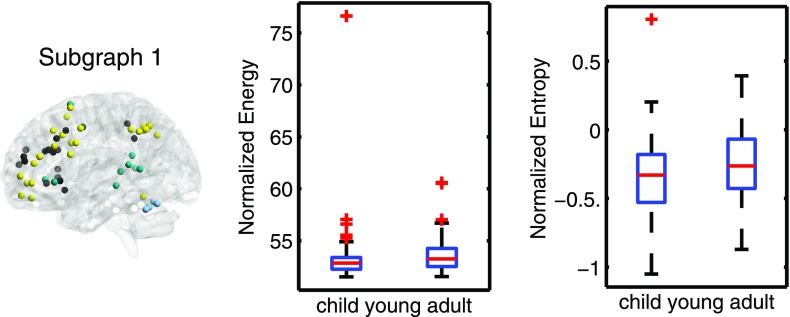
Age-related differences in subgraph expression. We observed significant differences between the two age groups in the standardized energy and entropy of the first subgraph, composed predominantly of regions in the frontal and parietal cortices subserving executive function (left). Standardized energy (middle) and standardized entropy (right) were both higher in the young adult group, suggesting higher levels of expression as well as a greater tendency to change expression level.

We observed significant differences between the groups in the expression and stability of the executive subgraph. Specifically, the standardized energy was higher in the group of young adults (Wilcoxon rank sum test: *z* = −2.72, *p* = 0.007; Figure 4A), suggesting that young adults have greater expression of systems involved in executive function. Similarly, standardized entropy was higher in the young-adult group (*z* = −2.34, *p* = 0.018; Figure 4B), suggesting a greater tendency in young adults than in children for this subgraph to change in its level of expression. Conversely, the executive subgraph in the younger subjects was less highly expressed, and less prone to change, potentially underscoring the presence of a less developed and less efficient executive system. These findings indicate a transition toward a greater precedence of executive networks and a greater switching of the regions and the interactions subserving these networks throughout development.

Importantly, these changes in the expression of the executive subgraph were associated with both developmental changes in behavior and individual differences in behavior above and beyond those expected with age. In the full sample of 780 individuals, we projected the subgraphs learned from the subset of 200 subjects onto each individual’s functional connectivity matrix to obtain temporal weights. We then examined the relationship between the executive subgraph weights and overall accuracy in the Penn Neurocognitive Battery (Moore, Reise, Gur, Hakonarson, & Gur, 2015). After regressing out the effect of age from both variables, we observed a statistically significant relationship between the flexibility of the executive subgraph weights and overall task accuracy on the battery (Gur et al., 2014), as compared to a null distribution of correlation coefficients (permutation test *p* < 0.01; see Supplementary Information, Chai et al., 2017). This finding suggests that greater flexibility in expression of executive regions supports individual differences in cognition.

## DISCUSSION

Using novel dynamic machine-learning techniques that decompose matrices into time-evolving functional subgraphs, we studied how dynamic patterns of connectivity mapped onto known cognitive systems and evolved with development in 200 subjects 8–11 and 19–22 years of age. We demonstrated that the majority of cognitive systems tend to act collaboratively over short timescales rather than independently. While sensorimotor systems displayed strong coupling to nearby regions, a number of systems related to executive function captured distributed interactions across the cortex. Moreover, we observed that high-energy subgraphs are more transient and flexible, while low-energy subgraphs are more stable in expression. Finally, we observed changes in the dynamics of an executive subgraph over development. As compared to the group of children, the group of young adults displayed an increase in the expression of the executive subgraph, as well as an increase in the switching behavior of the subgraph. These results complement previous studies with a new approach to examine state transitions among subgraphs as a continuous, overlapping process.

### Interactions Among Cognitive Systems

While correlated activity among distinct brain regions has been consistently observed during task execution, the evidence has shown that nontrivial correlations also exist as subjects lie quietly in a scanner—that is, in a so-called “resting state” (Fox et al., 2005). Moreover, relatively consistent patterns of distributed resting-state activity have been noted across neuroimaging methods (Deco, Jirsa, & McIntosh, 2011). Furthermore, resting-state brain networks have revealed architectures closely related to underlying anatomical connections (Deco & Corbetta, 2011) that dynamically change during passive activities in the absence of task demands (Andric & Hasson, 2015). The notion of globally coordinated (Zalesky, Fornito, Cocchi, Gollo, & Breakspear, 2014) and dynamically competing resting-state networks (Deco & Corbetta, 2011) directly motivates studies that can identify overlapping cognitive systems and can explain and predict their dynamics.

Previous work in spatially and temporally overlapping subgraph detection has uncovered cohesive structures in resting-state brain networks. Using a principal component analysis (PCA) approach, Leonardi et al. (2013) identified building blocks of dynamic networks that persist across time and subjects. Using another coactivation-pattern-driven approach, (Karahanoğlu & Van De Ville, 2015) showed that resting-state coactivation patterns display structures comparable to known resting-state networks. In contrast to these prior studies, we employed a nonnegative matrix factorization (NMF) approach (Kim & Park, 2007; Lee & Seung, 1999), which identifies subgraphs of a functional brain network that dynamically vary across subjects and across time in a parts-based manner, so that the connectivity for a subject in each time window is a nonnegative combination of basis subgraphs (Eavani, Satterthwaite, Gur, Gur, & Davatzikos, 2013). NMF addresses several assumptions made by PCA regarding the probabilistic structure of functional brain connectivity. First, functional brain networks are often described in terms of nonnegative, dynamic interactions between brain regions, with an intuitive interpretation that more positive interaction is related to stronger functional connectivity. Application of PCA to functional networks may yield positive or negative subgraph interactions and temporal expression coefficients, challenging neurophysiological interpretation. On the other hand, NMF enforces nonnegativity in the resulting subgraphs and temporal expression coefficients, which eases the interpretability of the relative expression of different subgraphs over time. Second, PCA assumes that subgraphs must be orthogonal, or nonoverlapping, a constraint that mandates that pairs of interacting brain regions can only be members of a single subgraph. The functional subgraphs uncovered in our study flexibly allow interacting brain regions to be members of more than one subgraph. Finally, PCA is limited to describing up to second-order statistics defined by the variance of functional connectivity strengths over time. Prior work has demonstrated that brain dynamics exhibit heavy-tailed probability distributions that can only be fully described with higher-order statistics (Buzsáki & Mizuseki, 2014). While the coactivation pattern technique employed by Karahanoğlu and Van De Ville does account for higher-order statistics, unlike NMF, their technique clusters the BOLD signal directly, rather than the dynamic functional network.

We observed salient connectivity patterns in resting-state fMRI data acquired from a group of subjects drawn from the Philadelphia Neurodevelopmental Cohort, where subgraphs corresponded directly to known cognitive systems (Figure 1A; Power et al., 2011) and captured salient dynamic interactions among them. Consistent with prior work, we found that sensorimotor regions, especially the visual cortex, tended to display strong connectivity within the system as well as strong local connections to nearby systems (Leonardi et al., 2013; Yeo et al., 2011). On the other hand, association regions involved in higher-level cognitive functions formed a number of integrated subgraphs (Figure 2B; Yeo et al., 2011). A specific example worth mentioning is the default mode network, which—while often considered distinctly separate from task-positive networks—has previously been observed to support attention and memory functions (Gu et al., 2015; Karahanoğlu & Van De Ville, 2015). We noted a similar pattern involving connections between the default mode and ventral attention systems. In contrast to a strict view of modularity, our results suggest that the brain employs a higher-dimensional architecture of widely integrated and spatially overlapping subgraphs at short timescales, rather than a set of “fully encapsulated” and functionally independent modules (Fodor, 1983). While a more simplistic view is that the brain consists of distinct subnetworks in opposition (e.g., task-positive and task-negative networks), increasing evidence supports the notion that such subnetworks of the brain may not be entirely separate (Allen et al., 2014).

### Dynamics of Resting-State Connectivity

It is now widely acknowledged that connectivity in the brain is not static. Instead, it dynamically varies on a timescale from minutes to days (Bassett et al., 2011; Bassett et al., 2013; Bassett et al., 2015). Switching patterns, in which a time of stable and highly correlated behavior rapidly changes to uncorrelated activity, with a sharp transition between states, have previously been noted in resting-state networks (Hansen, Battaglia, Spiegler, Deco, & Jirsa, 2015). We observed a striking correlation between the dynamic temporal behavior of subgraphs and their level of expression (Figure 3). For example, the visual cortex subgraph displayed low expression over time, captured by low energy, but also displayed consistent activity over time, captured by low entropy. In contrast, the executive subgraph, composed of cortical areas subserving executive functions, displayed highly variable activity and high expression over time.

The variance in subgraph flexibility that we observed has implications for our conceptualization of the roles of primary versus higher-order systems in the dynamics of cognition. Whereas visual and sensorimotor regions are relatively stationary, task control systems have more dynamic relationships (Power et al., 2011). In particular, the flexibility of the fronto-parietal network functions in maintaining adaptive online control and actively responding to feedback and task variety (Cole et al., 2013; Dosenbach et al., 2007). Dynamic reconfiguration of the fronto-parietal network has also been implicated in individual differences in executive function and enhanced memory performance (Braun et al., 2015). Here we captured continuous transitions in the dynamic switching behavior of subgraphs, rather than discrete state changes, yielding large fluctuations in the expression of frontoparietal regions, and slight fluctuations in the expression of sensorimotor regions.

### Neurodevelopmental Changes in the Executive System

Considerable changes in functional brain networks occur throughout neurodevelopment. The modularity of the brain’s structure evolves with age, as does interregional connectivity (Meunier, Achard, Morcom, & Bullmore, 2009; Supekar, Musen, & Menon, 2009). Functional connections within resting-state networks weaken with age, and connections among resting-state networks strengthen with age (Betzel et al., 2014). Furthermore, network modules become increasingly differentiated from childhood to late adolescence (Gu et al., 2015). Consistent with these findings, our results also suggest that functional network architecture undergoes significant changes in its fine-scale dynamics during neurodevelopment. We observed differences in the expression and temporal variability of the executive subgraph between young children and young adults (Figure 4). Specifically, the executive subgraph was more highly expressed in the young adults and displayed greater switching behavior, illustrating increasing flexibility of the fronto-parietal regions with age (Medaglia et al., 2015).

The delayed development of the frontoparietal regions is consistent with a recently posited trade-off between proactive cognitive control and creativity of thought. The prefrontal cortex is involved in complex cognitive functions, such as language, problem solving, switching attention, and decision making (Chrysikou, Weber, & Thompson-Schill, 2014). The transition from reactive control to proactive cognitive control during childhood also helps in suppressing unwanted memories and controlling impulses (Blackwell & Munakata, 2014). However, the delayed development of cognitive control confers certain advantages in childhood. Young brains are more flexible at updating beliefs and assimilating new information, because they are less biased by prior assumptions (Lucas, Bridgers, Griffiths, & Gopnik, 2014) and more hesitant to generalize rules (Chrysikou et al., 2014). As a result, young children are more adept at learning irregular patterns in linguistic conventions (Thompson-Schill, Ramscar, & Chrysikou, 2009) and using objects in creative and unconventional manners (Chrysikou, Novick, Trueswell, & Thompson-Schill, 2011). While the delayed emergence of frontal systems is disadvantageous in cognitive control, it is beneficial in terms of language acquisition, learning, and creativity in children (Blackwell & Munakata, 2014; Chrysikou et al., 2011; Medaglia et al., 2015).

### Methodological Considerations

A few methodological considerations are pertinent to this work. First, the matrix decomposition method used here imposed the constraint of positive subgraph edge weights and time-dependent coefficients, such that all subgraphs had some positive level of expression and the expression of subgraphs was additive. However, examining the relative expressions of a pair of subgraphs can convey the synergistic or antagonistic dynamics of these subgraphs.

Second, we recognize the analytic trade-offs inherent in large-scale applications of machine learning. Computational memory restricts the practical size of the concatenated, subject-level connectivity matrix, which grows as the number of subjects and the BOLD signal duration increases. Because nonnegative matrix factorization yields a low-rank approximation of the large connectivity matrix, increasing the number of subgraphs also increases the computational burden of storing and manipulating the subgraph and expression matrices. At the asymptotic limit of extracting the maximum number of subgraphs, the NMF problem is thought to be NP-hard (Vavasis, 2009). For these reasons, we were only able to study 200 subjects imaged as part of the PNC using the matrix decomposition algorithm.

Finally, we examined subgraphs from a population level (as was also done in Leonardi et al., 2013) and, using a previously established partitioning scheme of 264 nodes, characterized their expression during the resting state into cognitive systems. This work could be complemented in future by adjusting for individual variations in the system assignments, examining individual differences in subgraph structure and expression, and examining how the subgraphs and dynamics change during task conditions.

## CONCLUSION AND FUTURE DIRECTIONS

Here we developed a framework for uncovering overlapping and smoothly transitioning subgraphs that capture the interactions among cognitive systems and the changes in their expression. The subgraphs depicted varying patterns of cognitive system interactions, with sensory regions forming densely connected local subgraphs, and frontoparietal regions forming distributed and strongly interconnected subgraphs. More highly expressed subgraphs—those with higher energy—tended also to be more flexible in changing expression. From childhood to late adolescence, regions involved in executive function increased in their expression as well as in their dynamic switching behavior. Our results provide a context for understanding how neurocognitive processes evolve throughout normative development. In future work, it would be compelling to also study how different regions of the brain are recruited differently in neuropsychiatric disorders, and how the dynamic natures of the subgraphs differ. Neurodevelopmental disorders, such as schizophrenia, autism, and ADHD, impact network properties such as efficiency, path length, and modularity (Bassett & Bullmore, 2009; Bullmore & Sporns, 2012; Rudie et al., 2013). Applying a continuous, part-based method for identifying network subgraphs in disease contexts may yield interesting insights regarding any changes in network topology and dynamics in these contexts.

## METHODS

Data were collected in a collaboration between the Center for Applied Genomics at the Childrens Hospital of Philadelphia and the Brain Behavior Laboratory at the University of Pennsylvania. Resting-state functional MRI BOLD scans were acquired from 780 healthy children between the ages of 8 and 22. All scans were collected on a 3-tesla Siemens TIM Trio whole-body scanner with a 32-channel head coil. During the scan, the subjects fixated on a displayed crosshair while keeping their eyes open and remaining still for a duration of 6.2 min. The study procedures were approved by the Institutional Review Board of the University of Pennsylvania; all adults provided informed consent, and all guardians of minors provided informed consent. See Satterthwaite et al. (2014) for additional details regarding the imaging procedures.

Here we examined 100 subjects between 8.17 and 11.42 years of age (mean = 10.01; 49 male) and 100 subjects between 19.58 and 22.58 years of age (mean = 20.46; 44 male). We observed differences in motion (root-mean square [RMS] distance between consecutive scans, averaged over the full acquisition) between the two groups (mean in children = 0.078, mean in young adults = 0.060; two-sample *t*-test: *t*(198) = 3.22, *p* = 0.002). However, this difference was included as a nuisance variable in all analyses and was uncorrelated with the summary statistics of interest.

For each subject, we obtained the BOLD time series from each of *N* = 264 functional brain regions or network nodes. First we divided each BOLD time series into *T* = 51 sliding time windows, each 20 TRs in duration, with 90% overlap. Sliding windows of similar lengths, between 10 and 30 repetition times (TRs), have previously been used by other groups (Bassett et al., 2011; Braun et al., 2015; Leonardi et al., 2013). Next, we defined the functional connectivity, or edge weight, *A*_*ijl*_ as the wavelet coherence (see Supplementary Information, Chai et al., 2017) between the BOLD signals of node *i* and node *j* in each time window *l* in the frequency interval 0.02–0.08 Hz. The matrix **A** has dimensions *N* × *N* × *T*. We then divided each layer of **A** by the mean of that layer (Figures 1A–1C). See the Supplementary Information for further details regarding the BOLD signal preprocessing and motion correction.

Next, we unfolded each subject’s matrix **A** by taking the unique connections in each time window. That is, using the upper triangle of the *N* × *N* matrix in one time window (due to symmetry), we unfolded the matrix into an *N*(*N* − 1)/2 length vector. We then concatenated the vectors obtained from all *T* time windows. Therefore, for each subject, we obtained an *N*(*N* − 1)/2 × *T* matrix. We defined this matrix as a functional connectivity matrix **C**. We next regressed out the motion parameter (the temporal average of the RMS distances between consecutive scans) of the 200 subjects from each unique element of **A** and each of *T* time windows. In the functional connectivity matrix **C**, the unique elements correspond to the rows of **C**, and each time window corresponds to a column of **C**. Because the motion regression procedure resulted in a very small number of negative values in the connectivity matrices, which violated the nonnegativity constraint of the matrix decomposition algorithm, we set these values to zero.

In this study, we looked at the functional connectivity matrices of the 100 youngest and 100 oldest healthy and typically developing subjects imaged in the Philadelphia Neurodevelopmental Cohort. We concatenated these *S* = 200 matrices into matrix **X**, where **X** = [*C*_1_*C*_2_...*C*_200_]. This matrix **X** had dimensions *N*(*N* − 1)/2 × *TS*, where *N* = 264 nodes, *T* = 51 time windows, and *S* = 200 subjects (Figure 1D).

Nonnegative matrix factorization (NMF) is an unsupervised machine-learning technique (Lee & Seung, 1999) that factorizes the collection of functional connections in **X** into two matrices **W** and **H** that capture additive parts of the original set of dynamic functional networks, such that the matrix product **W** **H** ≈**X**. Here we employed the sparse NMF algorithm formulated in Kim & Park (2007):

$$\begin{array}{c} \min_{W,H}\frac{1}{2}\left\{∥X-WH{∥}_{F}^{2}+\eta ∥W{∥}_{F}^{2}+\beta \sum_{j=1}^{TS}∥H\left(:,j\right){∥}_{1}^{2}\right\},\\ \mathrm{such}\;\mathrm{that}\;W,\;H\geq 0\text{.} \end{array}$$

where **X** is the unfolded functional connectivity matrix, concatenated across time windows and subjects, **W** is a matrix of subgraph connectivity with size *N*(*N* − 1)/2 × *k*, and **H** is a matrix of time-dependent expression coefficients for each subject and subgraph with size *k* × *TS*. The parameter *k* determines the number of subgraphs obtained, *β* is a penalty parameter that enforces sparsity on the temporal expression coefficients, and *η* is a regularization parameter that provides an upper bound on the connection strengths within the functional subgraphs. To minimize the optimization problem, we applied the alternating nonnegativity constrained least squares until convergence (Žitnik & Zupan, 2012). We used the deterministic, nonnegative singular value decomposition (Boutsidis & Gallopoulos, 2008) to initialize the subgraph and expression coefficient matrices, **W** and **H**; this decomposition has shown rapid and stable convergence of the NMF algorithm.

To optimize the values for hyperparameters *k* and *β*, we computed the mean-squared reconstruction error through **X** −**W** **H** for a range of *k* and *β* values (see Supplementary Information, Chai et al., 2017, Figure S1), to obtain optimal values of *k* = 10 and *β* = 10^−2.0^. We observed that the reconstruction error was more sensitive to changes in *k* than in *β*, indicating that the results are robust to sparse temporal coefficients (see Supplementary Information, Figure S1). Similar to Kim & Park (2007), we set the parameter *η* to be the square of the maximal element in **X** to regulate the magnitude of connection strengths in the subgraphs described in **W**, which also depend on the range and level of sparsity of the temporal expression coefficients **H**.

We studied the roles of the cognitive systems in each subgraph by computing within-system and between-system connectivity. The 264 nodes were mapped to 13 well-known cognitive systems, including the visual, motor, auditory, default mode, salience, frontoparietal, cingulo-opercular, and attention systems (Power et al., 2011). For each *N* × *N* subgraph *V*, we defined the *within-system connectivity* as the mean strength of the functional connections within a cognitive system (Gu et al., 2015; Mattar et al., 2015): $R_{i}=\frac{\sum_{i,j\in P_{i}}V_{ij}}{|P_{i}{|}^{2}}$ where *P*_*i*_ is the set of nodes in a particular system, and |*P*_*i*_| is the number of nodes in the set. The *between-system connectivity* was defined as the mean strength of the functional connections between two distinct cognitive systems (Gu et al., 2015; Mattar et al., 2015): $I_{ij}=\frac{\sum_{i\in P_{i},j\in P_{j}}V_{ij}}{\left|P_{i}||P_{j}\right|}$, where *P*_*i*_ ≠ *P*_*j*_—that is, *P*_*i*_ and *P*_*j*_ are two different systems. Using within-system connectivity and between-system connectivity collapsed the 264-node × 264-node subgraph into a 13-system × 13-system matrix that summarized the interaction patterns of the 13 systems. Next, to measure how localized each subgraph was, we computed the spatial skewness of each one. More specifically, we unfolded the upper triangular of the 13 × 13 system-wide matrix (due to symmetry) into a column vector, omitting the matrix diagonal. We then computed the skewness of the distribution of elements in this column vector. Subgraphs with localized patterns of interaction have highly skewed distributions, while subgraphs with distributed patterns of interaction have less skewed distributions.

To visualize which cognitive systems were the most important contributors to each 13 × 13 subgraph (Figure 2B), we computed 1,000 permutations of the system labels of the 264 nodes and compared the connectivity of each true cognitive system (defined using the column sum of the 13 × 13 matrix) to the distribution of predicted connection strengths under the null hypothesis. We marked a cognitive system as significantly expressed in a subgraph if the strength of its connectivity in the subgraph was above the 95% confidence interval threshold (uncorrected for multiple comparisons), as defined by a nonparametric permutation distribution. This thresholding approach allowed us to determine the most highly expressed systems in each subgraph for visualization.

To capture the subgraphs’ dynamic changes in expression, we first computed the signal energy of the time-dependent coefficients for each subgraph and subject. Signal energy is defined as $\sum_{n=1}^{L}S_{n}^{2}$, where *S*_*n*_ is each time-dependent coefficient and *L* is the length of the signal for a subject. The signal energy quantified the level of expression for each component and subject over time windows. Second, we computed the signal entropy using a histogram-based entropy estimator method that computed the entropy as $\sum_{i=1}^{n}-P(x_{i})\log P(x_{i})$, where *P*(*X*) is a probability mass function computed using the histogram (Moddemeijer, 1989; Nelson et al., 2010). The signal entropy quantifies the dynamic switching behavior of subgraph expression.

To confirm the importance of the observed relationship between subgraph energy and entropy, we used a nonparametric statistical approach. We randomly permuted the elements in the time-dependent coefficient matrix, such that the permuted time-dependent coefficient matrix retained the same shape, but elements were shuffled at random across rows and columns in the permuted matrix. We then computed the correlation between energy and entropy in this shuffled, time-dependent coefficient matrix. This process was repeated for 1,000 permutations. We observed a significantly higher correlation between energy and entropy in the original signal than in the randomly permuted signals (permutation test: *p* < 0.001). These results confirmed that the observed relationship between the energy and entropy of subgraph expression was not expected under the null hypothesis, but instead represented an important neurophysiological process.

To faciliate between-subject comparisons, for each subgraph we standardized the energy and entropy values: We computed the means of the time-dependent coefficients for each subject and each subgraph, and then divided all time-dependent coefficients for each subject and each subgraph by their respective means. This corrected for differences in baseline expression that were not specific to individual subgraphs. In addition, because development is accompanied by an average change in head micromovements in the scanner, we sought to determine whether our results could be explained by this variable of noninterest. Note that we regressed out motion signals from the functional connectivity matrix before the matrix decomposition step. However, to be conservative, we also tested for relationships between motion and the two variables of interest: subgraph energy and entropy. We observed that energy and entropy were uncorrelated with subject motion (Pearson correlation coefficients: energy *r* = −0.104, *p* = 0.144; entropy *r* = −0.130, *p* = 0.068). These results confirmed that motion was unlikely to be an explanation for the observed differences in subgraph energy and entropy between the children and young adults.

The code for the full analysis pipelines is available at https://github.com/chail/NMF_neurodevelopment. The PNC data used in the analyses are publicly available through NIH dbGaP.

## ACKNOWLEDGMENTS

L.R.C. acknowledges support from the Rachleff Scholars Program. A.N.K. acknowledges support from the National Institutes of Health through award nos. R01-NS063039 and 1U24 NS 63930-01A1, the Citizens United for Research in Epilepsy (CURE) through a Julie’s Hope Award, and the Mirowski Foundation. D.S.B. acknowledges support from the John D. and Catherine T. MacArthur Foundation, the Alfred P. Sloan Foundation, the Army Research Laboratory and Army Research Office (contract nos. W911NF-10-2-0022 and W911NF-14-1-0679), the National Institute of Mental Health (2-R01-DC-009209-11), the National Institute of Child Health and Human Development (1R01HD086888-01), the Office of Naval Research, and the National Science Foundation (BCS-1441502 and BCS-1430087). The Philadelphia Neurodevelopmental Cohort imaging project was established by RC2 grants from the National Institute of Mental Health (MH089983 to REG). R.C.G. and R.E.G. were funded by grant nos. R01MH107235 and P50MH096891. T.D.S. was supported by grants K23MH098130 and R01MH107703; additional support was provided by the Dowshen Program for Neuroscience. Additional support to D.S.B. and T.D.S. was provided by the Institute for Translational Medicine and Therapeutics at the University of Pennsylvania.

## TECHNICAL TERMS

Matrix decomposition: a factorization of matrix **X** into two smaller-dimensional matrices **W**, **H** such that the matrix product **WH** approximates **X**.
Functional connectivity: A statistical inference of the pairwise interactions between brain regions over a period of time
Subgraph: a subset of network edges, whose strengths covary with time.
Subgraph expression: the amount a subgraph contributes to the observed network topology at a point in time, relative to other subgraphs.
Functional magnetic resonance imaging (fMRI): 3-D measurement of brain activity by detecting changes in blood oxygen content
Within-system connectivity: average of edge weights between nodes in the same cognitive system.
Between-system connectivity: average of edge weights between two sets of nodes in different cognitive systems.
Executive subgraph: collection of systems involving higher-order cognitive functions, such as fronto-parietal task control, salience, and ventral attention.
Energy: measurement of the dynamic intensity of subgraph expression, based on the sum of the squares of the weights.
Entropy: measurement of the average information conveyed, based on the distribution of expression weights over a period of time.
