# Supplementary material for: Evolution of brain network dynamics in neurodevelopment
Source: Netw Neurosci. 2017 Feb 1;1(1):14–30. doi: 10.1162/NETN_a_00001 (PMC6330215; doi:10.1162/NETN_a_00001)
Supplement: Supplementary file 1 [file netn-01-14-s001.pdf]

Supplementary Material for Evolution of Brain Network Dynamics in Neurodevelopment

Lucy R. Chai[1], Ankit N. Khambhati[1], Rastko Ciric[2], Tyler Moore[2], Ruben C. Gur[2], Raquel E. Gur[2], Theodore D. Satterthwaite[2], Danielle S. Bassett[1,3,4]

[1]*Department of Bioengineering, University of Pennsylvania, Philadelphia, PA 19104 USA*

[2]*Brain Behavior Laboratory, Department of Psychiatry, University of Pennsylvania, PA 19104 USA*

[3]*Department of Electrical & Systems Engineering, University of Pennsylvania, Philadelphia, PA 19104 USA*

[4]*Corresponding Author: dsb@seas.upenn.edu*

1 Supplementary Methods

Subjects The PNC was a collaboration between the Center for Applied Genomics at Childrens Hospital of Philadelphia (CHOP) and the Brain Behavior Laboratory at the University of Pennsylvania (Penn). Study procedures were approved by the Institutional Review Boards of both Penn and CHOP. The target population-based sample was of 10,000 youths who presented to the CHOP network for a pediatric visit and volunteered to participate in genomic studies of complex pediatric disorders [1]. A subsample of 1,445 participants, stratified by age and sex, were randomly selected for neuroimaging [2]. Of these, 1,275 had resting-state data acquired. Participants were excluded due to missing cognitive data, poor imaging data quality, or a history that suggested potential abnormalities of brain development. Data regarding medical history was gathered from both self-report at time of study entry as well as electronic medical records available from CHOP. Specifically, 234 participants were excluded due to a history of medical problems that might affect brain function, a history of inpatient psychiatric hospitalization, or current use of psychotropic medication. Additionally, 323 participants met exclusion criteria due to poor resting-state image quality [3–5], including if scan mean relative displacement exceeded 0.2mm (see below), if there were greater than 20 volumes with relative displacement greater than 0.25mm, or if gross between-run motion resulted in incomplete brain coverage. These exclusion criteria resulted in a final eligible pool of 780 participants aged 8–22 years (mean age 15.63 (SD=3.28); 333 males). Many participants were excluded due to multiple criteria. For computational reasons, of this full sample, we extracted the 100 youngest subjects and the 100 oldest subjects to be included in our study.

Image Acquisition All subject data were acquired on the same scanner (Siemens Tim Trio 3 Tesla, Erlangen, Germany; 32 channel head coil) using the same imaging sequences. Blood oxygen level dependent (BOLD) fMRI was acquired using a whole-brain, single-shot, multi-slice, gradient-echo (GE) echoplanar (EPI) sequence of 124 volumes with the following parameters: TR/TE=3000/32 ms, flip=90 degrees, FOV=192 $\times$ 192 mm, matrix=64 $\times$ 64, slice thickness/gap =3mm/0mm. The resulting nominal voxel size was 3.0 $\times$ 3.0 $\times$ 3.0 mm. A fixation cross was displayed as images were acquired. Subjects were instructed to stay awake, keep their eyes open, fixate on the displayed crosshair, and remain still. Prior to time-series acquisition, a 5-minute magnetization-prepared, rapid acquisition gradient-echo T1-weighted (MPRAGE) image (TR 1810 ms, TE 3.51 ms, FOV 180 $\times$ 240 mm, matrix 256 $\times$192, effective voxel resolution of 1 $\times$ 1 $\times$ 1mm) was acquired to aid spatial normalization to standard atlas space. Prior to scanning, in order to acclimate subjects to the MRI environment, a mock scanning session was conducted for each individual using a decommissioned MRI scanner and head coil. Mock-scanning was accompanied by acoustic recordings of the noise produced by gradient coils for each scanning pulse sequence. During these sessions, feedback regarding head movement was provided using the MoTrack (Psychology Software Tools, Inc, Sharpsburg, PA) motion tracking system. In order to further minimize motion, subjects' heads were stabilized in the head coil using one foam pad over each ear and a third over the top of the head. See [6] for the original description of imaging procedures.

Image Preprocessing Please see also a previous study describing the preprocessing framework [3]. Functional image processing used tools that are included in FSL [7] and AFNI [8]. Timeseries data was processed using a validated confound regression procedure that has been optimized to reduce the

influence of subject motion [3,4]. The first 4 volumes of the functional timeseries were removed to allow signal stabilization, leaving 120 volumes for subsequent analysis. Functional timeseries were band-pass filtered to retain frequencies between 0.01–0.08 Hz. Functional images were re-aligned using MCFLIRT [9]. Structural images were skull-stripped using BET [10]. Improved confound regression was performed using a 36-parameter model [11,12], which included 6 motion parameters as well as the temporal derivative, quadratic term, and temporal derivative of the quadratic of each and global signal regression [3,4]. Prior to confound regression, all confound parameters were band-pass filtered in an identical fashion as the timeseries data itself in order to prevent mismatch in the frequency domain and allow the confound parameters to best fit the retained signal frequencies [13]. Notably, as both spike regression and scrubbing have been found to bias graph properties, they were not applied here [12]. In addition, we regressed out motion from each element in the connectivity matrix to ensure that motion-related artifacts did not drive our functional-connectivity-based findings.

Processed subject-level BOLD images were co-registered to the T1 image using boundary-based registration [14] with integrated distortion correction as implemented in FSL 5 [7]. Whole-head T1 images were registered to the Montreal Neurologic Institute 152 1mm template using the top-performing diffeomorphic SyN registration that is part of ANTs [15–17]. All registrations were inspected manually and also evaluated for accuracy using spatial correlations. Network nodes were registered to subject space for timeseries extraction by concatenating the coregistration, distortion correction, and normalization transformations so that only one interpolation was performed in the entire process.

Functional Brain Network Construction We extracted regional mean BOLD time series from 264 functionally defined regions covering cortical and subcortical areas [18,19]. We apply a wavelet decomposition to the raw time series to extract information in the frequency interval $0.02 - 0.08$ Hz, using the Morlet wavelet transform (we use 0.02 Hz as the lower bound because our window duration is 60s). Wavelet-based methods have significant advantages in terms of denoising [20], robustness to outliers [21], and utility in null model construction [22]. Moreover, wavelet-based methods facilitate the examination of neurocognitive processes at different temporal scales without the edge effects in frequency space that accompany traditional band pass filters [23]. But perhaps the most compelling argument in support of wavelets [24] derives from the fact that cortical fMRI time series display slowly decaying positive autocorrelation functions (also known as long memory) [25,26]. This feature undermines the utility of measuring functional connectivity between a pair of regional time series using a correlation (time domain) or coherence (frequency domain), because both time- and frequency-domain measures of association are not properly estimable for long memory processes [27]. In contrast, wavelet-based methods provide reliable estimates of correlation between long memory time series [28,29] derived from fMRI data [24,30,31] Based on these advantages, wavelet-based estimates of functional connectivity have provided extensive insights into brain network organization in health [21], aging [24], neurological disorders [32], sleep [33], and cognitive performance [34].

Consistent with prior work [35,36], we estimated functional connectivity A_{ijl}^{raw} between any two pair of regions i and j in layer l using a wavelet coherence [37]. A wavelet coherence can be thought of as a localized correlation coefficient in time frequency space[37]. Traditionally, a coherence is

often reported as the mean squared coherence between time series over a large frequency interval. Similarly, the wavelet coherence is the mean squared coherence estimated over a large frequency interval in the wavelet domain [37]. As such, the wavelet coherence has a minimum possible value of 0 and a maximum possible value of 1, in contrast to a Pearson correlation coefficient which has a minimum possible value of -1. We chose to apply a coherence measurement over a correlation measurement based on prior work demonstrating its usefulness in the context of fMRI neuroimaging data [38], and based on the parsimony of network modeling methods that deal with positive-only edge weights [35,39], necessary for the non-negative matrix decomposition procedure.

The fully weighted adjacency matrix $\mathbf{A}^{\mathrm{raw}}$ therefore represents the functional brain network for a given subject in which network nodes represent brain regions and network edges represent functional connections between those regions. Following [40], we divide each layer of a subject's functional connectivity matrix by the average value of that layer to obtain the normalized functional connectivity matrix $\mathbf{A}_{\mathrm{l}} = \frac{\mathbf{A}_{\mathrm{l}}^{\mathrm{raw}}}{<\mathbf{A}_{\mathrm{l}}^{\mathrm{raw}}>}$ which we use for the remainder of our analysis.

To further assure that individual differences in motion did not drive our functional connectivity findings, we regress motion out of each element in the functional connectivity matrix. Finally, note that following these preprocessing choices, we do not apply any arbitrary thresholds to the functional connectivity matrix; instead, the structure of the full connectivity matrix is explored in an effort to remain sensitive to small variation in connectivity patterns tracking with development.

2 Supplementary Results

Matrix decomposition optimization parameters Given a functional connectivity matrix X, non-negative matrix factorization factors the matrix into a basis matrix of subgraphs, W, and a matrix of time-dependent coefficients, H, such that $X \approx WH$. In order to determine the optimal decomposition of the functional connectivity matrix X, we performed a grid-search procedure over a parameter k, that sets the number of subgraphs, and β, that controls the sparsity of the time-coefficient matrix H. We computed the reconstruction error, defined as $||X - WH||_F$, over all pairs of k and β. Based on our finding that RSS is more dependent on k than β, we assessed the average RSS over all β for each k. Intuitively, the addition of each additional subgraph progressively decreases the explanatory power of that subgraph, yielding an RSS curve that decays with increasing k. Based on the first-order difference of RSS *versus* k, we selected $k = 10$, a cutoff which has also been selected in prior literature [41,42]. Next, we selected $\beta = 10^{-2.0}$ by considering the minimum of the curve obtained from plotting the reconstruction error against β, while fixing $k = 10$. The minimum point ensures that we obtain a reconstruction that is as close as possible to the original matrix (Fig 1).

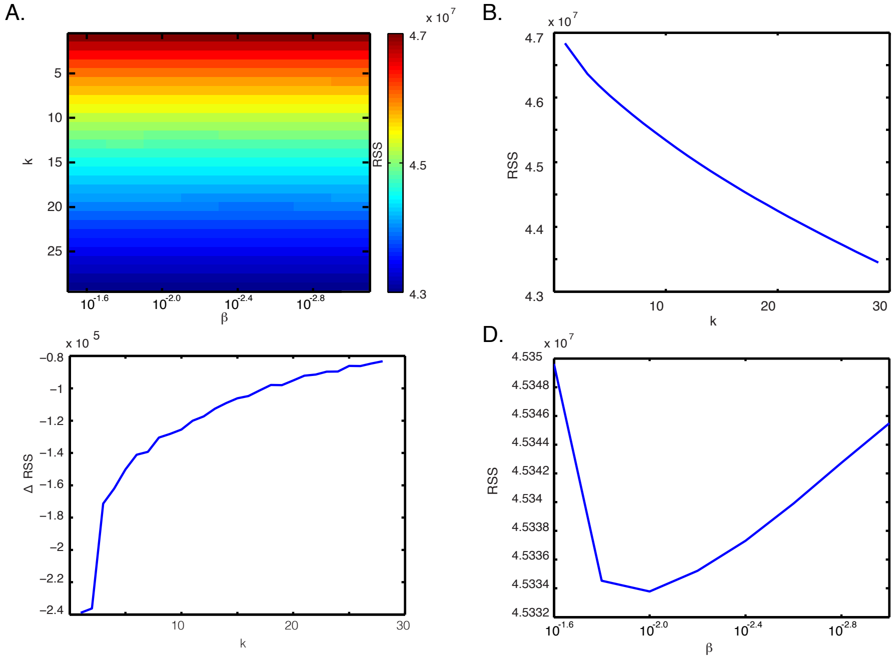

Figure 1: **Grid Search of Optimal Parameters.** *(A)* Computing reconstruction error using the residual sum of squares in the range $k = [1...29]$, $\beta = [10^{-1.6}...10^{-3}]$. *(B)* We plotted the reconstruction error *versus* k, the number of subgraphs. Each additional component reduces the reconstruction error. Because the reconstruction error depends more heavily on the number of subgraphs rather than the parameter β, we averaged across values of β for this plot. *(C)* We then plotted the derivative of the reconstruction error vs. k and selected $k = 10$ in accordance with prior literature [41,42]. *(D)* Plotting reconstruction error against β for $k = 10$. We selected β to be the minimum of the curve.

Subgraphs obtained from matrix decomposition From the NMF decomposition of the functional connectivity matrix, we obtained a set of 10 subgraphs. In the main text, we investigate the cognitive systems expressed in each subgraph. Here, we present the same subgraphs across 264 brain regions rather than 13 cognitive systems. We observed that the subgraphs captured varying interactions among brain regions. Some subgraphs captured distributed interactions across the en-

tire network, while other subgraphs captured fairly localized interactions. These results suggest that the brain consists of a complex landscape containing both regions with distributed activity patterns and regions with more localized activity (Fig. 2).

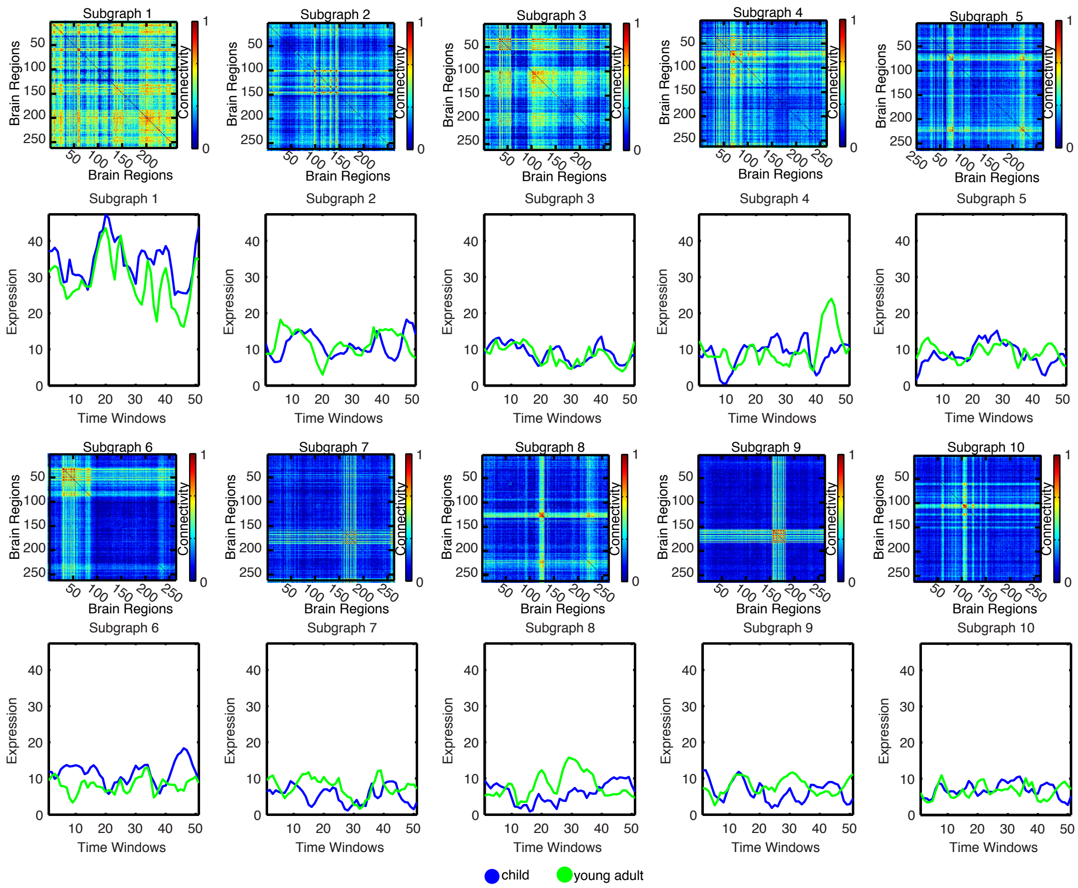

Figure 2: **NMF Decomposition of Functional Connectivity Matrix.** Subgraphs obtained from applying NMF to the concatenated functional connectivity matrix, ordered according to decreasing average expression. We normalized the colorbar to between 0 and 1. We also show the temporal coefficient weights for a representative child subject (in blue) and a representative young adult subject (in green).

Alternate metrics of temporal stability To measure the changes in subgraph expression using the temporal coefficients, we computed the entropy of the coefficients for each subject and each subgraph. Intuitively, entropy is related to the unpredictability of information context. Thus, subgraphs that have a greater tendency to change in expression should have higher entropy (i.e. their time courses are more unpredictable), and subgraphs that are stable in expression should have low entropy. However, because the entropy measure is based on the distribution of the temporal coefficients, it is not dependent on the precise ordering of the temporal coefficients. As an alternative method of measuring the changes in subgraph expression over time, we computed a temporal derivative metric, which is defined as the absolute value of the first order difference between adjacent temporal coefficients. This metric directly captures the changes in subgraph expression levels over time. We observed that the entropy metric was highly correlated to the temporal derivative metric (Pearson's correlation coefficient $r = 0.99$, $p < 0.001$). This suggests that, while entropy is purely based on the distribution of the temporal coefficients, the underlying distribution is related to the fact that there is temporal structure, as measured by the temporal derivative (Fig. 3).

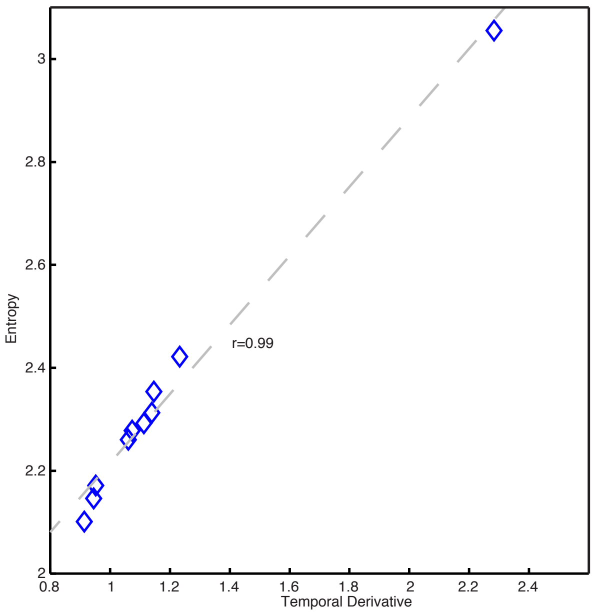

Figure 3: **Metric of temporal stability.** We observe that the entropy of the subgraph temporal coefficients is highly correlated to the temporal derivative of the subgraph temporal coefficients.

Temporal Null Model To investigate whether the fluctuations in the time-dependent coefficients were meaningful rather than random, we constructed null models of the time-dependent coefficients. To that end, for each subgraph and each subject, we permuted the time-dependent coefficients measuring the expression of the subgraph over time windows at random 1000 times (i.e., permuting the order of the 51 time-dependent coefficients for each subject and subgraph). We then computed the temporal derivative and the entropy of each permuted signal, and averaged across subgraphs and permutations to obtain one statistic per subject corresponding to temporal derivative, and one statistic per subject corresponding to entropy. Similarly, we computed the temporal derivative and the entropy in the real data to also obtain one statistic per subject corresponding to temporal derivative, and one statistic per subject corresponding to entropy. We observed

that the temporal derivative is significantly higher in the permuted signals than the real signals ($t_{398} = -80.59$, $p < 0.001$). However, because entropy is a histogram based estimator, permuting the order of the signals does not affect its underlying distribution. Thus, there was no difference between the permuted signals and the real signals ($t_{398} = 0$, $p = 1$); Fig. 4).

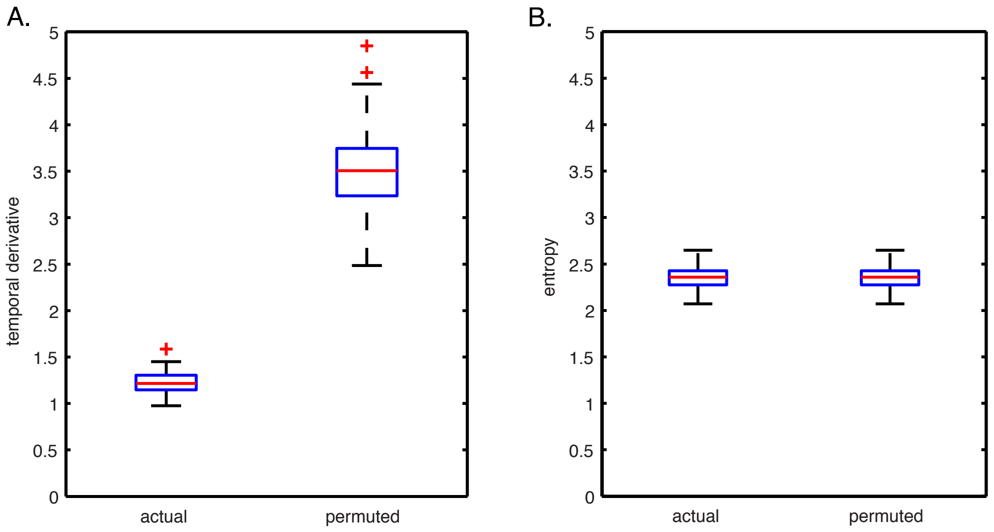

Figure 4: **Null model of temporal coefficients.** To determine the difference between the observed fluctuations in the temporal coefficients and random fluctuations, we constructed temporal null models by permuting temporal coefficients. We note that this leads to significant changes in the temporal derivative, but it does not change the entropy due to the distribution-based nature of the entropy metric.

Robustness of cognitive systems We next asked whether the connectivity patterns of the cognitive systems we observed using 10 subgraphs would change significantly if the number of subgraphs were increased or decreased. Applying the same matrix decomposition, we first selected $k = 8$ subgraphs rather than $k = 10$ subgraphs (Fig. 5). Similar to the main text, we ordered the subgraphs in decreasing average expression. We observe that the structure of the subgraphs remains largely similar to the structure of the original 10 subgraphs. Moreover, we observe similar neurodevelopment effects in the first subgraph capturing brain regions involved in executive function.

Next, we applied the same procedure using $k = 12$ (Fig. 6), and arranging subgraphs in order of decreasing average expression. At this point, we observe that the executive subgraph referred to in the main text breaks apart into multiple subgraphs. However, the structure of other subgraphs remains consistent between $k = 12$ and $k = 10$.

We then selected $k = 45$ (Fig. 7), arranging subgraphs in order of decreasing average expression. We observe some similarities in connectivity patterns, but also breakage of the original 10 subgraphs into multiple subgraphs. Moreover, we also observe a number of noisy and less coherent subgraphs, particularly as the subgraph number increases. We note the the ordering of the subgraphs differs than that of the main text: this may be due to the splitting of the original 10 subgraphs into multiple subgraphs, such that each of the split subgraphs has a lower weight in the temporal coefficients than the original joint subgraph.

Lastly we aimed to the the robustness of the results to changes in the parameter β. Recall that β controls the sparsity of the time-dependent coefficients matrix H. To that end, we performed

the same matrix decomposition procedure as described in the main text, but set the parameter $\beta = 0$ (Fig. 8). We obtain similar subgraph structure, however the ordering of the subgraphs has changed. We note that modifying the parameter β redistributes the temporal weights, increasing the magnitude of the temporal coefficients for the first subgraph while decreasing the magnitude of temporal coefficients for the remaining subgraphs.

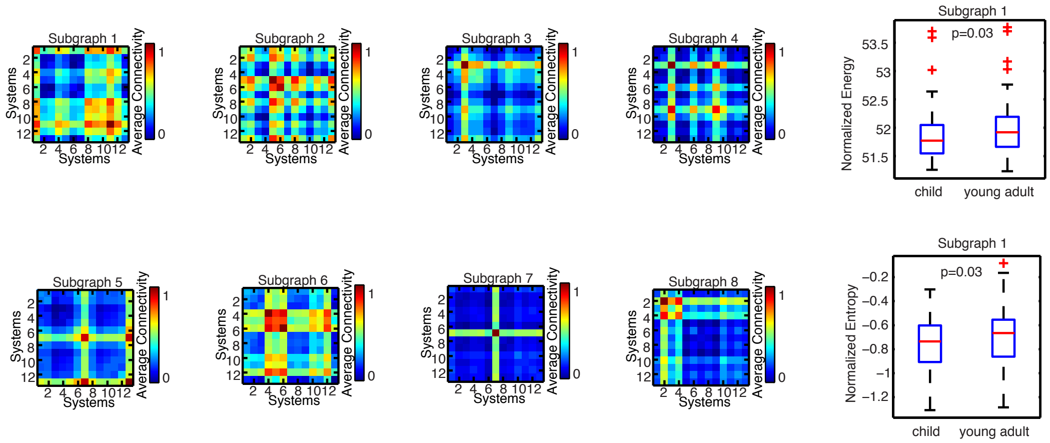

Figure 5: **Matrix decomposition using 8 subgraphs.** We computed system-wide connectivity over 8 subgraphs, normalizing the colorbar between 0 and 1. We observe that the structure of these subgraphs remains largely similar to the original 10 subgraphs. Moreover, we obtain similar results when comparing the energy and entropy of the first subgraph of executive regions between the group of children and young adults.

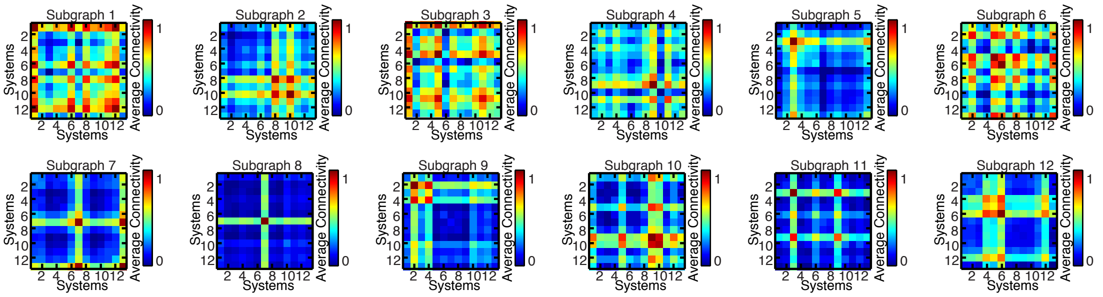

Figure 6: **Matrix decomposition using 12 subgraphs.** We computed system-wide connectivity over 12 subgraphs. We normalized the colorbar to between 0 and 1. We observe that some subgraphs retain a similar structure as the original 10 subgraphs, while some subgraphs (namely Subgraph 1 in the main text), breaks apart into multiple subgraphs.

Neurodevelopmental effects in the remaining subgraphs In the main text, we hypothesized that the energy and entropy of the first subgraph, capturing a number of cognitive systems related to executive function, would change over development. Here, we show the results for the remaining subgraphs (Fig. 9). A Bonferroni correction was not applied because the executive subgraph was first tested due to our hypotheses.

Flexibility and individual differences in behavior We aimed to determine the relationship between subject performance on a neurocognitive battery and the temporal coefficients for the subgraph capturing cognitive systems involved in executive function. To that end, we first obtained the overall accuracy scores [43] from the Penn Neurocognitive Battery [44,45] for each of 779 subjects (one subject was omitted due to not having an accuracy score; and cognitive scores from the first visit were used for subjects with whom we performed repeated testing). To obtain these overall accuracy scores, we fit a unidimensional factor model and observed that – while all tests contributed at

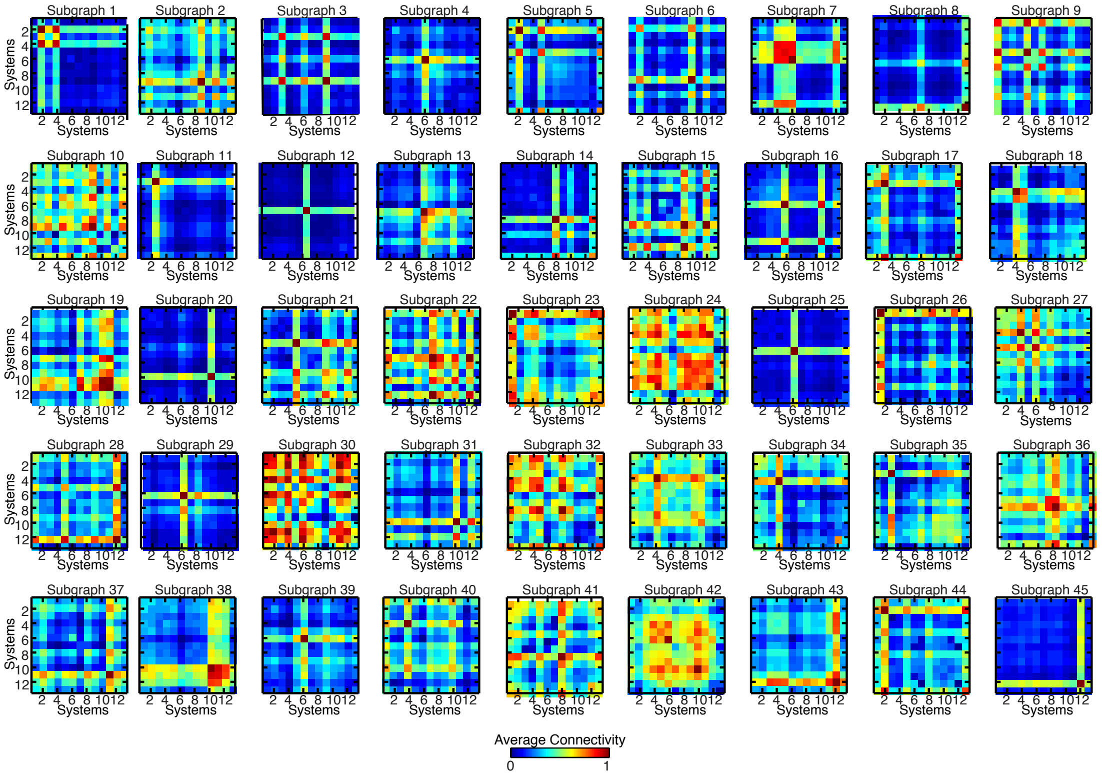

Figure 7: **Matrix decomposition using 45 subgraphs.** We computed system-wide connectivity over 45 subgraphs, normalizing the colorbar between 0 and 1. Again, we observe that increasing the number of subgraphs tends to break apart some of original 10 subgraphs, although we observe some similarities between the 45 subgraphs and the original 10 subgraphs.

least moderately – the most highly-contributing tests were the verbal reasoning, matrix reasoning, and emotion differentiation tests; the least contributing tests were the emotion identification, word memory, and visuo-spatial memory tests. For a similar factor analysis on a subset of the data, see [43], Table 5.

We note that while computation memory limits the size of a concatenated functional connectivity matrix, limiting our analyses to 200 subjects, no such restriction exists when decomposing

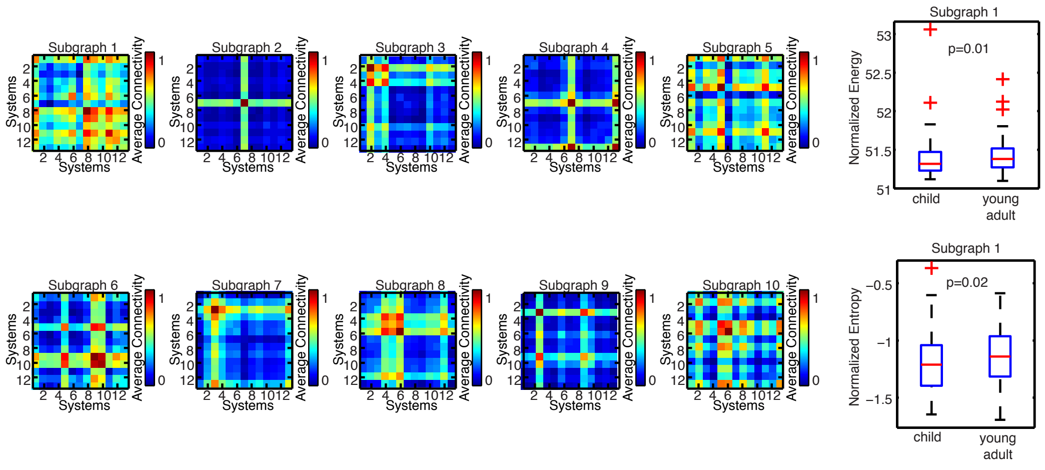

Figure 8: **Robustness to changes in beta.** We repeated the same matrix decomposition procedure as described the main text, but set the parameter $\beta = 0$. We computed system-wide connectivity over the 10 subgraphs, normalizing the colorbar between 0 and 1.

each subject's functional connectivity matrix individually. Thus, we obtained temporal weights, H, for the 779 subjects by computing a non-negative least squares regression to optimize the equation: $C = WH$, where C is each subject's functional connectivity matrix and W is the subgraphs matrix from the 200 subjects studied in the main text. Intuitively, while the subgraphs matrix W was learned from a subset of 200 subjects, it represents a mathematical basis set for functional brain networks uniformly sampled from a subject pool with a broad age distribution. Therefore, this back-projection method quantifies how highly each of the original subgraphs is expressed in the entire set of 779 subjects. We then normalized the executive subgraph temporal coefficients, as was done in the main text (dividing each subject's coefficients by their respective mean), and computed the temporal derivative of the normalized coefficients.

Because cognitive abilities increase markedly as youth develop, it is possible that these re-

sults could be driven simply by neurodevelopment, and not be a direct marker of individual differences in cognition. To determine whether the relationship between the temporal derivative of the executive subgraph coefficients and the accuracy score was independent of age effects, we regressed out the effects of both motion and age on the cognitive scores and on the temporal weights. We then computed a null distribution of Pearson's correlation coefficients between the temporal derivative and overall accuracy scores, using 1000 permutations of the temporal weights. From this non-parametric permutation approach, we observed a significant difference between the observed correlation between the overall accuracy score and the temporal derivative of the executive subgraph, in comparison to the null distribution (permutation test, $p < 0.01$). These results suggest that greater flexibility in expression of executive regions supports individual differences in cognition.

Variations on motion regression In the main text, we regressed out the motion parameter (the temporal average of RMS distance between consecutive scans) from every element of the functional connectivity matrix, as was done in prior work [46]. Here, we regressed out the motion parameter on the temporal coefficients matrix H obtained from matrix decomposition, rather than on the elements of the functional connectivity matrix. More specifically, we regressed out the motion statistic for 200 subjects from corresponding temporal coefficients across subjects, and repeated this process for each of 10 subgraphs and 51 time windows. We obtain similar results using this motion regression procedure, observing that both energy and entropy of an executive function-related subgraph increase with age.

Custom Templating In order to avoid registration bias and maximize sensitivity to detect regional effects that can be impacted by registration error, a custom template was created with ANTs [47]; T1 images were normalized to this population-specific template space using the top-performing SyN diffeomorphic registration method implemented in ANTs [48]. Structural images were then processed with 'antsCorticalThickness' [49], which uses the custom template to guide brain extraction, N4 bias correction [50], and Atropos probabilistic tissue segmentation [51]. This custom template was used in combination with the following motion censoring approach.

Motion censoring As high motions measurements may affect functional connectivity metrics, we repeated the same analyses as describe in the main text after motion censoring was applied. Iterative censoring is the most current scrubbing technique developed by Power et al. (2014) [52]. Whereas the original scrubbing approach discarded high-motion volumes only after processing was completed, iterative censoring acts to minimize the impact of these volumes at every stage of the processing pipeline. For instance, high-motion time points are not taken under consideration during detrending or fitting steps, and a Lomb-Scargle transform interpolates over noisy data so that it does not impact adjacent volumes during temporal filtering. Here, a high-motion volume was defined as any volume with relative RMS motion in excess of 0.25. Ultimately, high-motion volumes are discarded.

In the main text, we note that the first subgraph obtained from matrix composition captured a number of cognitive systems involved in executive function. After motion censoring, as well as using a custom template, we note a similar pattern of interactions emerges in the first subgraph.

We observed a visually similar looking first subgraph, and a significant correlation between these two patterns of interaction (Pearson's correlation coefficient $r = 0.53$, $p < 0.001$). Furthermore, we observe similar trends in the energy of the first subgraph, with energy higher in the group of young adults than the group of children (Wilcoxon rank-sum test $z = -3.08$, $p = 0.002$). While we observe higher entropy in the group of young adults than the group of children, this difference is no longer significant (Wilcoxon rank-sum test $z = -1.10$, $p = 0.27$), and may be due to changes in the underlying distribution of the temporal coefficients after high-motion volumes are discarded. We note that a limitation to the motion censoring procedure is the changes in the number of time windows. Thus, subjects each contribute a different number of observations to the matrix factorization step.

Independent Sampling In the main text, we selected the 100 youngest subjects (ages 8.17–11.42) and the 100 oldest subjects (ages 19.58–22.58) among the 780 subjects imaged in the PNC, due to limitations in computational memory. Here, we repeated the same procedure using an independent subset of 200 subjects (100 aged between 11.42–13.33 and 100 aged between 18.17–19.50, Fig. 12). We observed a pictorially similar subgraph decomposition among the regions in the executive subgraph (Pearson's correlation coefficient $r = 0.81$, $p < 0.001$ between the original executive subgraph and the executive subgraph in the independent sample.) Furthermore, we observed a consistent trend in the increase of subgraph entropy with development (Wilcoxon rank-sum test $z = -2.16$, $p = 0.03$). While we observed higher energy in the older group of subjects in this sample as well, the difference is no longer significant (Wilcoxon rank-sum test $z = -1.88$, $p = 0.06$) and may be due to the decreased range of ages in the 200 subjects used in this sample.

References

1. Gur, R. C. *et al.* Age group and sex differences in performance on a computerized neurocognitive battery in children age 8-21. *Neuropsychology* **26**, 251–265 (2012).

2. Satterthwaite, T. D. *et al.* Neuroimaging of the philadelphia neurodevelopmental cohort. *Neuroimage* **86**, 544–553 (2014).

3. Satterthwaite, T. D. *et al.* An improved framework for confound regression and filtering for control of motion artifact in the preprocessing of restingstate functional connectivity data. *Neuroimage* **64**, 240–256 (2013).

4. Satterthwaite, T. D. *et al.* Heterogeneous impact of motion on fundamental patterns of developmental changes in functional connectivity during youth. *Neuroimage* **83C**, 45–57 (2013).

5. Satterthwaite, T. D. *et al.* Impact of in-scanner head motion on multiple measures of functional connectivity: Relevance for studies of neurodevelopment in youth. *Neuroimage* **60**, 623–632 (2012).

6. Satterthwaite, T. D. *et al.* Neuroimaging of the philadelphia neurodevelopmental cohort. *Neuroimage* **86**, 544–553 (2014).

7. Jenkinson, M., Beckmann, C. F., Behrens, T. E. J., Woolrich, M. W. & Smith, S. M. FSL. *Neuroimage* **62**, 782–790 (2012).

8. Cox, R. W. AFNI: Software for analysis and visualization of functional magnetic resonance neuroimages. *Comput Biomed Res* **29**, 162–173 (1996).

9. Jenkinson, M., Bannister, P., Brady, M. & Smith, S. Improved optimization for the robust and accurate linear registration and motion correction of brain images. *Neuroimage* **17**, 825–841 (2002).

10. Smith, S. M. Fast robust automated brain extraction. *Hum Brain Mapp* **17**, 143–155 (2002).

11. Friston, K. J., Williams, S., Howard, R., Frackowiak, R. S. & Turner, R. Movement-related effects in fMRI time-series. *Magn Reson Med* **35**, 346–355 (1996).

12. Yan, C.-G. *et al.* A comprehensive assessment of regional variation in the impact of head micromovements on functional connectomics. *Neuroimage* **76C**, 183–201 (2013).

13. Hallquist, M. N., Hwang, K. & Luna, B. The nuisance of nuisance regression: spectral misspecification in a common approach to resting-state fMRI preprocessing reintroduces noise and obscures functional connectivity. *Neuroimage* **82C**, 208–225 (2013).

14. Greve, D. N. & Fischl, B. Accurate and robust brain image alignment using boundary-based registration. *Neuroimage* **48**, 63–72 (2009).

15. Avants, B. B., Epstein, C. L., Grossman, M. & Gee, J. C. Symmetric diffeomorphic image registration with cross-correlation: evaluating automated labeling of elderly and neurodegenerative brain. *Med Image Anal* **12**, 26–41 (2008).

16. Avants, B. B. *et al.* A reproducible evaluation of ANTs similarity metric performance in brain image registration. *Neuroimage* **54**, 2033–2044 (2011).

17. Klein, A. *et al.* Evaluation of 14 nonlinear deformation algorithms applied to human brain MRI registration. *Neuroimage* **46**, 786–802 (2009).

18. Power, J. D. *et al.* Functional network organization of the human brain. *Neuron* **72**, 665–678 (2011).

19. Nelson, S. M. *et al.* A parcellation scheme for human left lateral parietal cortex. *Neuron* **67**, 156–170 (2010).

20. Fadili, M. & Bullmore, E. A comparative evaluation of wavelet-based methods for hypothesis testing of brain activation maps. *NeuroImage* **23**, 1112–1128 (2004).

21. Achard, S., Salvador, R., Whitcher, B., Suckling, J. & Bullmore, E. A resilient, low-frequency, small-world human brain functional network with highly connected association cortical hubs. *The Journal of neuroscience* **26**, 63–72 (2006).

22. Breakspear, M., Brammer, M. J., Bullmore, E. T., Das, P. & Williams, L. M. Spatiotemporal wavelet resampling for functional neuroimaging data. *Human brain mapping* **23**, 1–25 (2004).

23. Percival, D. B. & Walden, A. T. Wavelet methods for time series analysis (cambridge series in statistical and probabilistic mathematics) (2000).

24. Achard, S. & Bullmore, E. Efficiency and cost of economical brain functional networks. *PLoS Comput Biol* **3**, e17 (2007).

25. Maxim, V. *et al.* Fractional gaussian noise, functional mri and alzheimer's disease. *Neuroimage* **25**, 141–158 (2005).

26. Wink, A. M., Bernard, F., Salvador, R., Bullmore, E. & Suckling, J. Age and cholinergic effects on hemodynamics and functional coherence of human hippocampus. *Neurobiology of aging* **27**, 1395–1404 (2006).

27. Beran, J. *Statistics for long-memory processes*, vol. 61 (CRC press, 1994).

28. Whitcher, B., Guttorp, P. & Percival, D. B. Wavelet analysis of covariance with application to atmospheric time series. *Journal of Geophysical Research* **105**, 941–962 (2000).

29. Gençay, R., Selçuk, F. & Whitcher, B. J. *An introduction to wavelets and other filtering methods in finance and economics* (Academic press, 2001).

30. Bullmore, E. *et al.* Wavelets and functional magnetic resonance imaging of the human brain. *NeuroImage* **23**, S234–S249 (2004).

31. Achard, S., Bassett, D. S., Meyer-Lindenberg, A. & Bullmore, E. Fractal connectivity of long-memory networks. *Physical Review E* **77**, 036104 (2008).

32. Supekar, K., Menon, V., Rubin, D., Musen, M. & Greicius, M. D. Network analysis of intrinsic functional brain connectivity in alzheimer's disease. *PLoS Comput Biol* **4**, e1000100 (2008).

33. Spoormaker, V. I. *et al.* Development of a large-scale functional brain network during human non-rapid eye movement sleep. *The Journal of neuroscience* **30**, 11379–11387 (2010).

34. Gießing, C., Thiel, C. M., Alexander-Bloch, A. F., Patel, A. X. & Bullmore, E. T. Human brain functional network changes associated with enhanced and impaired attentional task performance. *The Journal of Neuroscience* **33**, 5903–5914 (2013).

35. Bassett, D. S. *et al.* Dynamic reconfiguration of human brain networks during learning. *Proc Natl Acad Sci U S A* **108**, 7641–7646 (2011).

36. Bassett, D. S. *et al.* Task-based core-periphery organization of human brain dynamics. *PLoS Comput Biol* **9**, e1003171 (2013).

37. Grinsted, A., Moore, J. C. & Jevrejeva, S. Application of the cross wavelet transform and wavelet coherence to geophysical time series. *Nonlin Processes Geophys* **11**, 561–566 (2004).

38. Sun, F. T., Miller, L. M. & D'Esposito, M. Measuring interregional functional connectivity using coherence and partial coherence analyses of fMRI data. *NeuroImage* **21**, 647–658 (2004).

39. Bassett, D. S. *et al.* Task-based core-periphery organization of human brain dynamics. *PLoS Comput. Biol* **9**, e1003171 (2013).

40. Saad, Z. S. *et al.* Correcting brain-wide correlation differences in resting-state fMRI. *Brain Connect* **3**, 339–352 (2013).

41. Leonardi, N. *et al.* Principal components of functional connectivity: a new approach to study dynamic brain connectivity during rest. *NeuroImage* **83**, 937–950 (2013).

42. Eavani, H. *et al.* Identifying sparse connectivity patterns in the brain using resting-state fmri. *Neuroimage* **105**, 286–299 (2015).

43. Moore, T. M., Reise, S. P., Gur, R. E., Hakonarson, H. & Gur, R. C. Psychometric properties of the Penn Computerized Neurocognitive Battery. *Neuropsychology* **29**, 235–246 (2015).

44. Gur, R. C. *et al.* Computerized neurocognitive scanning: I. Methodology and validation in healthy people. *Neuropsychopharmacology* **25**, 766–776 (2001).

45. Gur, R. C. *et al.* A cognitive neuroscience-based computerized battery for efficient measurement of individual differences: standardization and initial construct validation. *Journal of Neuroscience Methods* **187**, 254–262 (2010).

46. Gu, S. *et al.* Emergence of system roles in normative neurodevelopment. *Proceedings of the National Academy of Sciences* **112**, 13681–13686 (2015).

47. Avants, B. B. *et al.* A reproducible evaluation of ants similarity metric performance in brain image registration. *Neuroimage* **54**, 2033–2044 (2011).

48. Klein, A. *et al.* Evaluation of 14 nonlinear deformation algorithms applied to human brain mri registration. *Neuroimage* **46**, 786–802 (2009).

49. Tustison, N. J. *et al.* Large-scale evaluation of ants and freesurfer cortical thickness measurements. *Neuroimage* **99**, 166–179 (2014).

50. Tustison, N. J. *et al.* N4itk: improved n3 bias correction. *Medical Imaging, IEEE Transactions on* **29**, 1310–1320 (2010).

51. Avants, B. B., Tustison, N. J., Wu, J., Cook, P. A. & Gee, J. C. An open source multivariate framework for n-tissue segmentation with evaluation on public data. *Neuroinformatics* **9**, 381–400 (2011).

52. Power, J. D. *et al.* Methods to detect, characterize, and remove motion artifact in resting state fmri. *Neuroimage* **84**, 320–341 (2014).

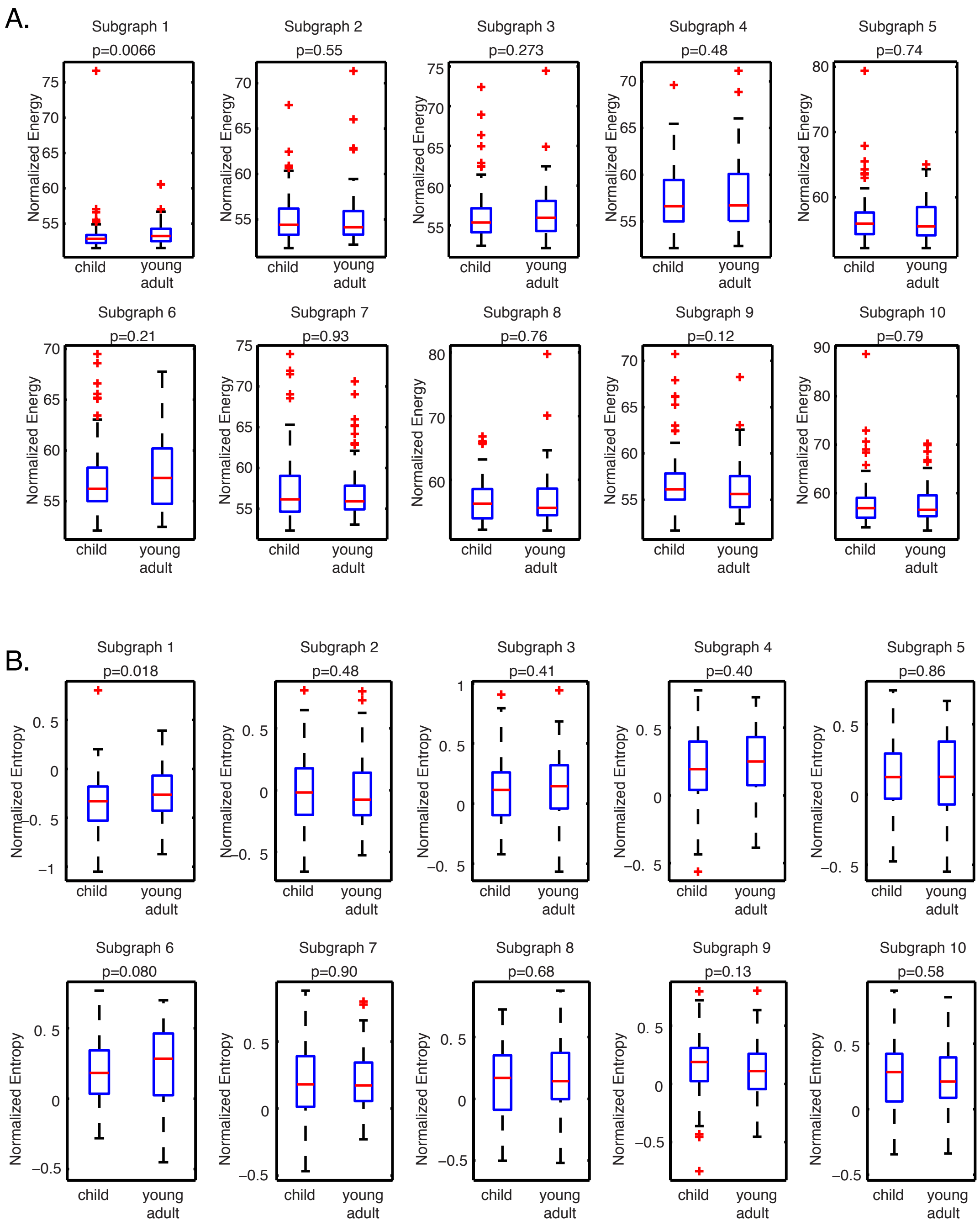

Figure 9: **Neurodevelopmental changes in energy and entropy** *(A)* Normalized energy over all 10 subgraphs for the child and young adult groups. *(B)* Normalized entropy over all 10 subgraphs for the child and young adult groups.

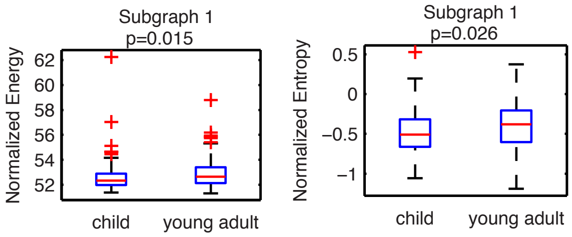

Figure 10: **Motion regression on temporal coefficients** We obtain similar results regarding energy and entropy trends in neurodevelopment after regressing the motion parameter from the temporal coefficients.

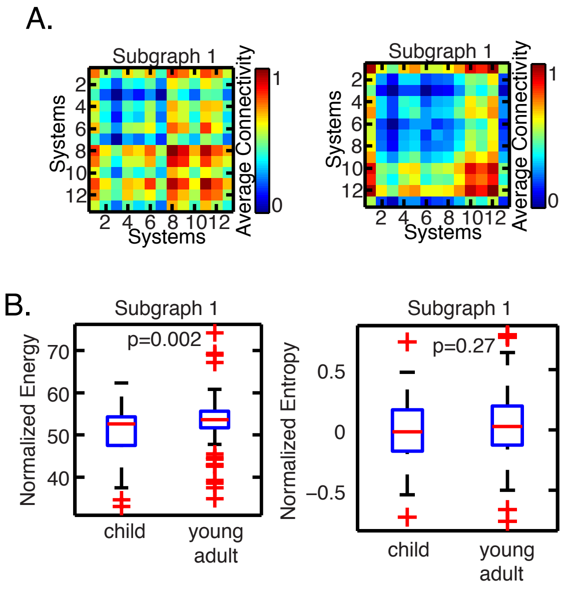

Figure 11: **Motion Censoring** *(A)* The first subgraph, capturing regions involved in executive function in the main text *(left)*, and the first subgraph after motion censoring *(right)*. The colorbar is normalized between 0 and 1. *(B)* Boxplots showing energy and entropy distributions among the group of children and the group of young adults after motion censoring was applied.

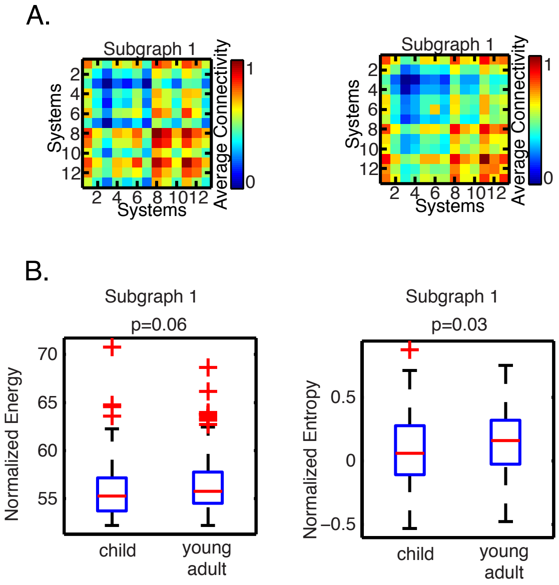

Figure 12: **Independent Sampling.** *(A)* The first subgraph, capturing regions involved in executive function in the main text with subject ages 8–11 and 19–22 *(left)*, and the first subgraph using an independent sample of 200 subjects among the 780 subjects in the PNC with ages 11–13 and 18–19. *(right)*. The colorbar is normalized between 0 and 1. *(B)* Boxplots showing energy and entropy distributions between the group of 100 younger (ages 11-13) subjects and the group of 100 older (ages 18-19) subjects.
